# Healthcare-associated urinary tract infections in urology

**DOI:** 10.3205/id000074

**Published:** 2021-08-30

**Authors:** José Medina-Polo, Kurt G. Naber, Truls E. Bjerklund Johansen

**Affiliations:** 1 Department of Urology, Health Research Institute i+12, Hospital Universitario 12 de Octubre, Madrid, Spain; 2 Department of Urology, Technical University of Munich, Germany; 3 Institute of Clinical Medicine, University of Oslo, Norway

**Keywords:** antibiotic resistance, healthcare-associated urinary tract infection (HAUTI), multidrug-resistance organism (MDRO), urinary catheter, urology department

## Abstract

The purpose of the present review is to report the incidence and characteristics of healthcare-associated urinary tract infections (HAUTIs) in urology with their microbiological and resistance patterns. Urinary tract infections are the main type of healthcare-associated infection in patients hospitalized in a urology ward. Patients admitted to urology departments report a high prevalence of urinary tract catheterization, up to 75% during the hospitalization period, and up to 20% had a urinary catheter before admission. An endourological surgical procedure is another risk factor for HAUTIs. Other risk factors for HAUTIs are the presence of immunosuppression and previous urinary tract infections.

In urological patients, Enterobacterales are the principal causative agent of HAUTIs, and *E. coli* is the most frequently isolated microorganism. However, there is also a high rate of microorganisms other than *E. coli* such as *Klebsiella *spp. and *Enterococcus *spp. Non-*E. coli* microorganisms show a higher prevalence in immunosuppressed patients and those with urinary catheters before admission. High resistance patterns are reported in patients with HAUTIs, and ESBL-producing bacteria are frequently described. Moreover, the isolation of multidrug-resistant microorganisms is more common in immunosuppressed patients, those with previous urinary tract infections, and urinary catheters into the upper urinary tract. Treatment must be tailored according to patient characteristics and patient profiles, bearing in mind the ORENUC classification for risk factors (no risk factors (O), recurrent urinary tract infections risk factors (R), extraurogenital risk factors (E), nephropathic disease (N), urological risk factors (U), permanent urinary catheter and non-resolvable urological risk factors (C)).

## Introduction

A healthcare-acquired infection (HAI) is defined as a localized or systemic condition that results from the action of an infectious agent or its toxin. HAIs include infections that occur when the patient is receiving healthcare, and it must not be present at the time of hospitalization. Thus, HAIs usually appear more than 48 hours after hospitalization [1]. The term ‘HAI’ not only relates to infections acquired during hospitalization, but also includes those who meet any of the following criteria: patients receiving intravenous therapy or specialized treatment of a wound at home; patients on hemodialysis; those receiving intravenous chemotherapy; or have been hospitalized in an acute care facility for two or more days in the past three months. Finally, those people who are institutionalized in residences or long-stay centers are also included.

HAIs show differentiating characteristics concerning other types of infections. First, the microbiological pattern involved in community-acquired infections is different from that shown by HAIs. Some microorganisms are typically considered nosocomial, such as *Pseudomonas aeruginosa* [1]. In the case of urinary tract infections (UTIs), differences are observed among community-acquired (CAUTIs) and healthcare-acquired UTIs (HAUTIs). On the one hand, the most frequently isolated microorganism is *Escherichia coli* (*E. coli*), which represents up to 70–80% of the pathogens isolated in positive cultures; this percentage is below 50% in the case of urinary tract infections related to healthcare [2]. Furthermore, HAIs are potentially serious complications in hospitalized patients and lead to increased costs and higher mortality rates [3], [4]. Increase in costs is due to an increase in medical requirements and a more extended hospital stay. It is estimated that an episode of urinary tract infection is associated with a prolonged hospital stay from 1 to 3 days [5], [6]. The term ‘urinary tract infection’ traditionally includes asymptomatic bacteriuria and symptomatic lower urinary tract infection. Moreover, febrile UTI, pyelonephritis, renal or perirenal abscess are also included as urinary infections and considered major infections. Other types of infection are infections of the male accessory glands such as acute orchitis or prostatitis. This category also includes urinary tract infections in patients with urinary catheters [2].

A key point in the management of healthcare-associated urinary tract infections (HAUTIs) is the necessity to prescribe antibiotic treatment as early as possible. Different studies have shown that a longer evolution time in septic patients before starting specific antibiotic treatment is associated with higher mortality. It has even been reported that start of adequate antibiotic treatment within the first hour from the clinical appearance is associated with better outcome in terms of morbidity and mortality [7], [8]. This fact underlines the importance of knowing the microbiological spectrum and resistance patterns in the local area related to the type of infections according to patient characteristics. Therefore, patient profiles can be created, and local protocols can be developed accordingly to achieve the highest success of treatment. This fact must go hand in hand with a judicious choice of antibiotics so as not to cause an increase in resistance by using broad-spectrum antibiotics indiscriminately.

Patients admitted to a Urology Service have an increased risk of developing HAUTIs. They frequently undergo some type of surgical procedure during hospitalization, and a high percentage are carriers of a urinary catheter both before and during admission [9], [10], [11]. Sometimes both risk factors are present since the type of urological surgery most frequently carried out is through an endourological transurethral access. Performing transurethral surgery entails, in practically all cases, the need to use a urinary catheter [12]. Therefore, the most common type of HAIs in urology is going to be UTI. However, these types of infections do not only affect urological patients. Globally, UTIs account for 20% to 40% of infections developed during hospitalization [6], [13]. For patients hospitalized in a urology unit, the percentage of HAUTIs rises to 60–70% of all HAIs [14].

The objective of the present review is to give an overview of HAUTIs by analyzing published data regarding incidence, associated risk factors, microbiological profiles and antibiotic resistance of the different microorganisms.

## Methods

A comprehensive search and review of the literature was carried out with focus on studies evaluating HAUTIs in patients admitted to urology. The PubMed library was searched with the terms ‘HAUTI’ or ‘healthcare-associated infections’ or ‘hospital acquired infections’ or ‘nosocomially-acquired infections’ and ‘urology’. Forty-five records were identified and reviewed. Furthermore, the references of included articles were reviewed in order to include all relevant records. For patients with catheter in the upper urinary tract, the PubMed library was searched using the terms ‘double J stent’ or ‘nephrostomy tube’ and ‘infections’ and ‘urology’. Urinary tract infections (UTIs) are defined according to the recommendations of the Infectious Diseases Society of America (IDSA). A urinary tract infection is defined by the presence of ≥10^5^ CFU (colony-forming units)/ml of a bacterial species isolated in a urine culture in a patient with symptoms suggestive of UTI as this is the definition used in most of the studies included in our review [15], [16]. However, ≥10^3^ CFU/ml of a bacterial species isolated in a urine culture in a patient with symptoms suggestive of UTI is currently accepted as definition [2], [17]. In patients with a urinary catheter and symptoms compatible with urinary tract infection the accepted cut-off point is ≥10^3^ CFU/ml [18]. Asymptomatic bacteriuria is defined as the presence of ≥10^5^ CFU/mL of one bacterial species isolated in a urine culture in two consecutive samples in the absence of signs suggestive of UTI [2]. Asymptomatic bacteriuria does not require to be treated with antibiotics, except before manipulations of the urinary tract and during pregnancy.

Multidrug-resistance was defined according to the ECDC and CDC definitions of multidrug resistance (MDR), extensive drug resistance (XDR) and pan-drug resistance (PDR) [19]. This classification was used in the GPIU study (Global Prevalence Study on Infections in Urology) [20]. MDR was defined as acquired non-susceptibility to at least one agent in three or more antimicrobial categories, XDR was defined as non-susceptibility to at least one agent in all but two or fewer antimicrobial categories (i.e. bacterial isolates remain susceptible to only one or two categories), and PDR was defined as non-susceptibility to all agents in all antimicrobial categories [19].

We review the incidence of HAUTIs and their risk factors in urological patients. We also analyze the microbiological characteristics and resistance patterns. Patient profiles have been defined based on comorbidities and urological factors such as having a urinary catheter either before or during admission, making a distinction for the type of catheter (urethral catheter, double J stent, nephrostomy tube) according to the ORENUC phenotyping [2], [21], [22].

## Results

### Incidence

**Figure 1:**
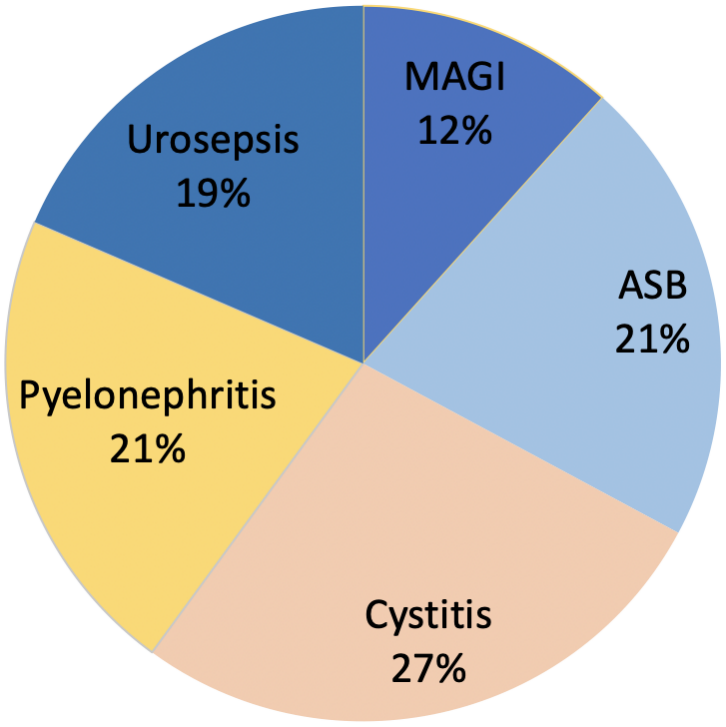
Type of HAUTIs according to the GPIU study 2003–2013 [29]; MAGI: male accessory gland infection, ASB: asymptomatic bacteriuria

Healthcare-related infections (HAIs) are a significant cause of concern for healthcare providers. For this reason, governmental, scientific and medical organizations have proposed and implemented different measures to prevent the development of this type of infection. Among them, at the international level, the work of the National Nosocomial Infection Surveillance System (NNISS) [4] should be highlighted. On the other hand, observational studies evaluating HAIs are widely carried out in intensive care units such as the ENVIN group who has been reviewing these types of infections for more than twenty years [23]. At the hospital level, the EPINE working group annually reviews the prevalence of HAIs in the different hospital units [6], [13]. This is a study of patients who are admitted to each of the hospital units at a given time point, collecting the prevalence of HAIs and their characteristics [13]. However, there are few published studies carried out in the urological setting. We therefore highlight the work carried out by the working group of the EAU Section of Infections in Urology (ESIU) belonging to the European Association of Urology, which for almost 20 years has been reviewing HAIs in urological patients. The project is called GPIU [20], [24], [25], [26]. According to the results of the Pan European Prevalence (PEP) study and Pan Euro-Asian Prevalence (PEAP) study, the incidence of HAIs in urology units was found to lie between 5% and 14% [5], [27]. According to the GPIU study, 27% of infections are cystitis and 21% pyelonephritis. Moreover, 19% of HAUTIs presented as sepsis (Figure 1 (Fig. 1)) [20], [28], [29]. The incidence of infections may be reduced with the implementation of a protocol which monitors the incidence of HAIs, and includes awareness among medical staff, nurses, patients and their relatives [30].

### Risk factors for HAUTIs

The differential characteristics of urological patients have been analyzed in studies such as the one by Cullen et al. [10] reviewing the microbiological characteristics of urinary tract infections, community-acquired and hospital-acquired, for 11 years and in an independent group for those from urology. In this group of patients, a higher incidence of stones, anatomical abnormalities of the urinary tract and lower urinary tract symptoms (LUTS) was observed. They are also patients with greater exposure to antibiotics due to previous recurrent UTIs and urological instrumentation. All these reasons imply that urological patients show a higher rate of antimicrobial resistance [10], [31]. Moreover, patients admitted to a urology ward report a high prevalence of urinary tract catheterization, up to 72% during the hospitalization period. On the other hand, 18% of the patients had a urinary catheter before admission. Surgery with an endourological approach is another risk factor for infections as it is performed in 54.5% of patients admitted in urology [32]. Due to the high percentage of patients with a urinary catheter, urinary tract infection is the most frequent type of HAIs in a urology ward, 70% of the total number of HAIs. The percentage increases even more in patients undergoing endourological transurethral surgery, in which 95.2% of HAIs are urinary tract infections [9], [32]. Reviews carried out in other hospital units, including medical and surgical wards, reported that urinary tract infections represent 15% to 57% of HAIs [28], [33]. In addition to the specific risk factors of urological patients previously mentioned, there are other factors classically described as older age, nutritional status (decreased albumin), anemia, immunosuppression, diabetes mellitus, connective tissue diseases, and lifestyle factors such as smoking, obesity and alcoholism [14]. 76.9% of the patients admitted to the urology ward present at least one risk factor, and the percentage rises to 97% in those with HAIs. The presence of immunosuppression and the existence of a previous urinary tract infection are associated with a higher risk of HAUTIs during admission [32].

Risk factors may be classified according to the ORENUC classification which takes into account host risk factors. The following phenotypes are defined: no known risk factors (O), recurrent urinary tract infections risk factors (R), extraurogenital risk factors (E), nephropathic disease (N), urological risk factors (U), permanent urinary catheter and non-resolvable urological risk factors (C). Therefore, the ESIU recommends evaluating HAIs according to the clinical presentation, the severity grade, host risk factors (ORENUC) and pathogen risk factors such as identity and antibiotic susceptibility of the causative pathogen [2], [21]. All factors must be borne in mind in a comprehensive risk assessment before empirical treatment, especially in severe infections [22].

### Microbiological patterns

**Figure 2:**
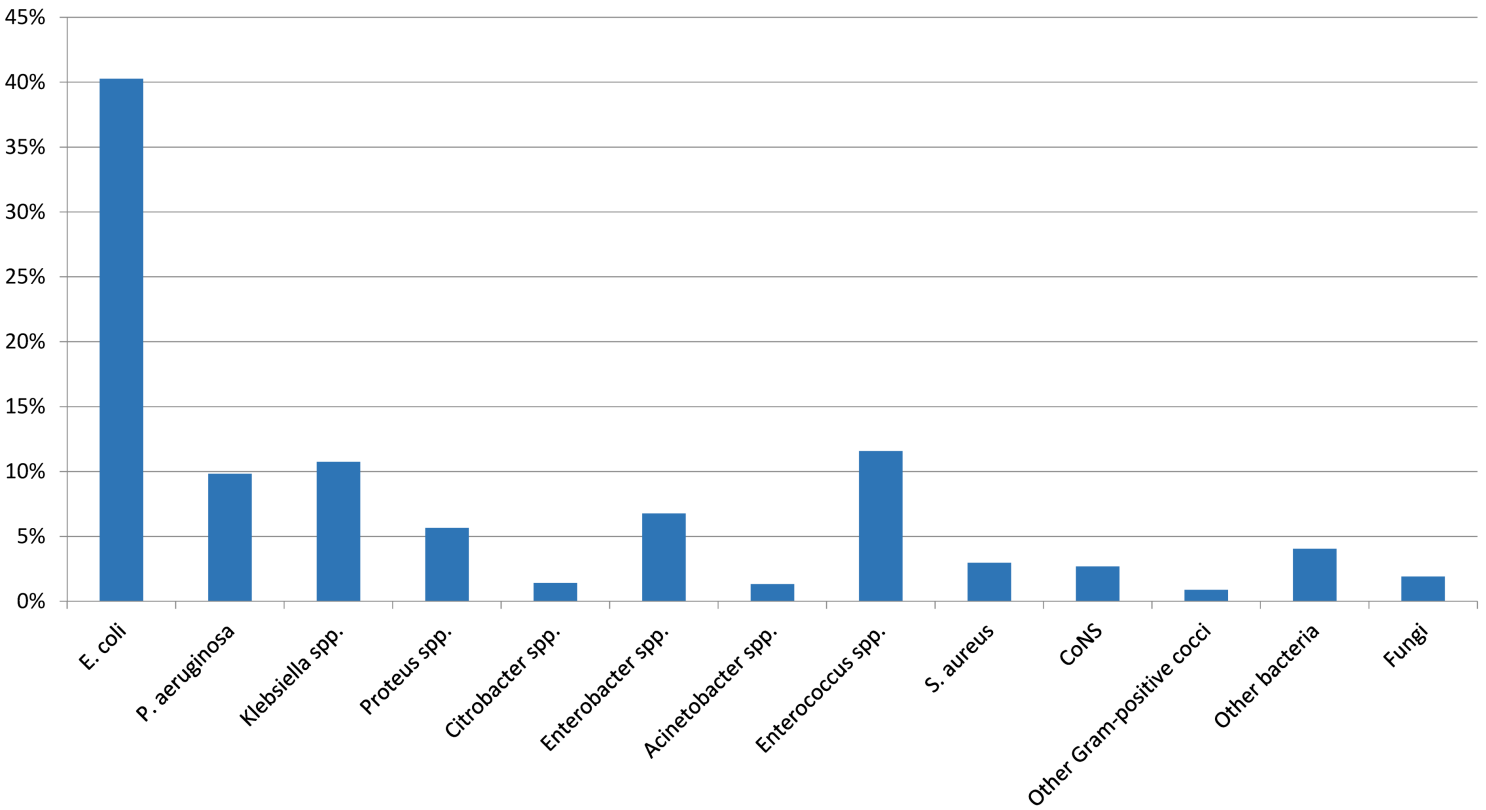
Distribution of organisms in patients with HAUTIs including in the GPIU study 2003–2013 [29]; CoNS: coagulase-negative staphylococci

**Figure 3:**
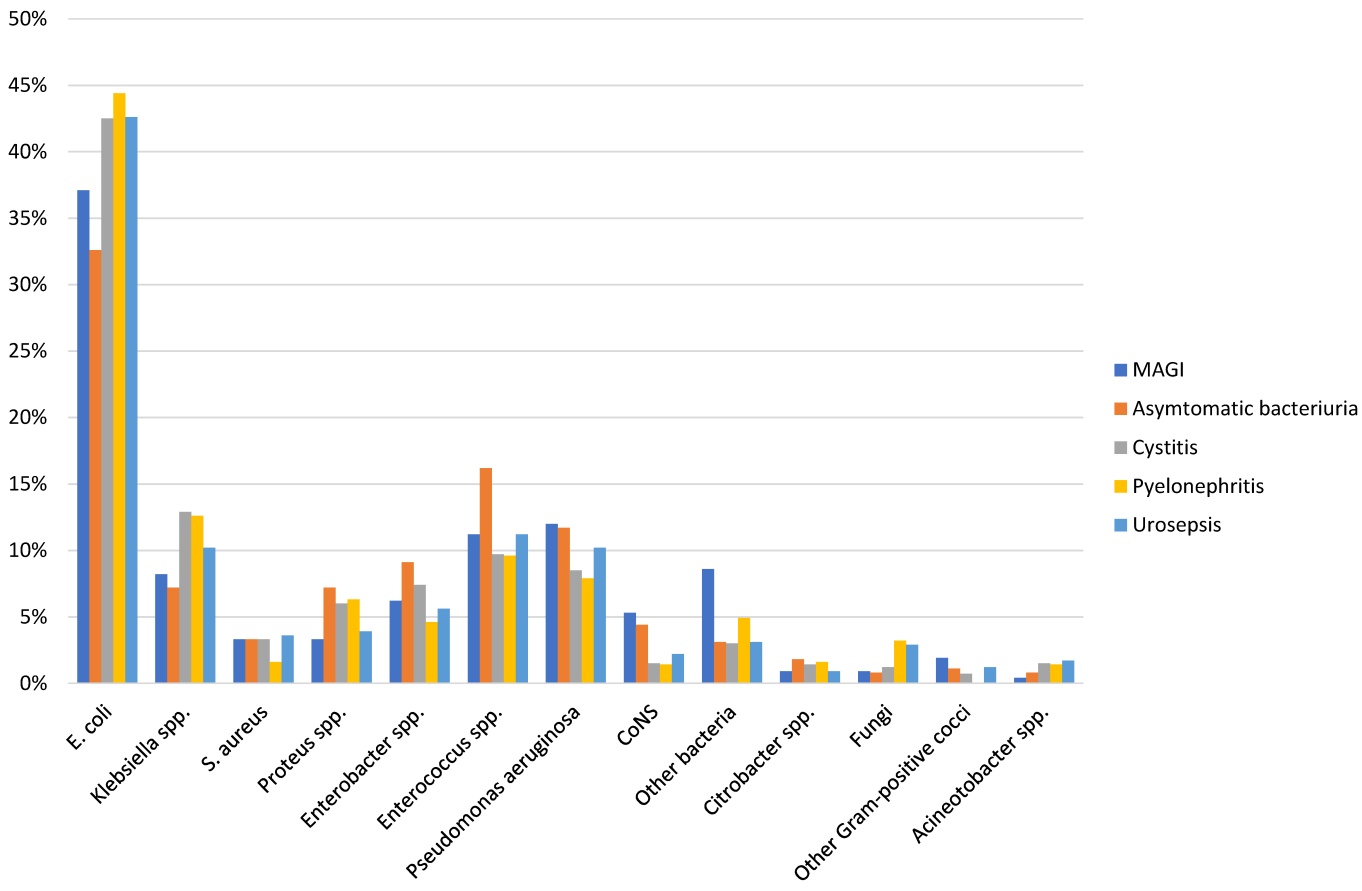
Distribution of organisms isolated according to the type of HAUTI in the GPIU study 2003–2013 [29]; MAGI: male accessory gland infection, ASB: asymptomatic bacteriuria, CoNS: coagulase-negative staphylococci

In urological patients, *Enterobacterales* are the main causative agents of HAUTIs. According to data from the GPIU group, *E. coli* is the most frequently isolated microorganism in cultures, representing percentages above 40% [5], [28], [29] (Figure 2 (Fig. 2)). Table 1 (Tab. 1) shows the distribution of organisms according to the type of infection and its origin for different published series. *Staphylococcus epidermidis* is usually isolated as contamination. *Candida* spp. isolation is mainly related to colonization in patients with multiple microorganisms’ isolation and being treated with antibiotics [9]. Figure 3 (Fig. 3) summarizes the microorganisms isolated according to the type of HAUTI. Nosocomially-acquired urinary tract infections have a lower prevalence of *E. coli* in comparison with community-acquired. Besides, various factors have been described that are related to a higher prevalence of other pathogens than *E. coli*. For example, in the case of urinary tract infections in older patients, it is more common to isolate *Enterobacterales* other than *E. coli* [34]. UTI in the previous months is also a risk factor for *Klebsiella* spp. isolation and it can be represented in 62.5% of positive cultures [9]. Bacteria such as *Enterococcus* spp. have been described with a higher prevalence in immunosuppressed patients and those with urinary catheters before admission [35], [36]. In the case of urological patients, higher rates of resistance are related to the high prevalence of urinary diversion catheter and urinary tract instrumentation [37], [38]. According to the data published by the GPIU group, resistance to quinolones and second-generation cephalosporins is up to 50% [26], [39]. Table 2 (Tab. 2) summarizes the rates of resistance of *E. coli* in reports published from different regions. Within Europe, the highest resistances are observed in Mediterranean countries [25], [40], [41]. It is necessary to highlight the high percentages of resistance to quinolones with figures ranging from 35% to 57%. These high levels of antibiotic resistance have been related to the wide use of this pharmacological group in recent years. Thus, it has been shown that the countries with the highest per capita prescription of antibiotics are those with the highest rates of resistance. Similarly, a reduction in the prescription of a group of antibiotics can be correlated with a decrease in antibiotic resistance [42].

In the case of infections caused by *Klebsiella* spp., the main differential characteristic is that they show higher resistance rates than *E. coli* isolates. Data are available from different geographical areas (Table 3 (Tab. 3)).

*Pseudomonas aeruginosa* is a pathogen that shows high rates of resistance, 36% for piperacillin/tazobactam, 30% for carbapenems and 55% for quinolones (Table 4 (Tab. 4)) [25], [39], [43].

*Enterococcus* spp. represent the second most frequently isolated bacterial species in some studies [32]. Broader use of cephalosporins and fluoroquinolones may explain the reason why *Enterococcus* spp. are more frequently isolated [44]. Both groups of antibiotics have moderate or no efficacy against *Enterococcus faecalis* and *Enterococcus faecium*, while amoxicillin has usually shown a good susceptibility profile. *Enterococci* are a species of microorganisms with a specific susceptibility profile due to exchange of genetic elements related to the development of resistance. In case of resistance to the most commonly used antibiotics, vancomycin is an effective alternative, but must be reserved for cases with isolation of *Enterococcus faecium* (Table 5 (Tab. 5)) [2], [20].

### Resistance patterns

**Figure 4:**
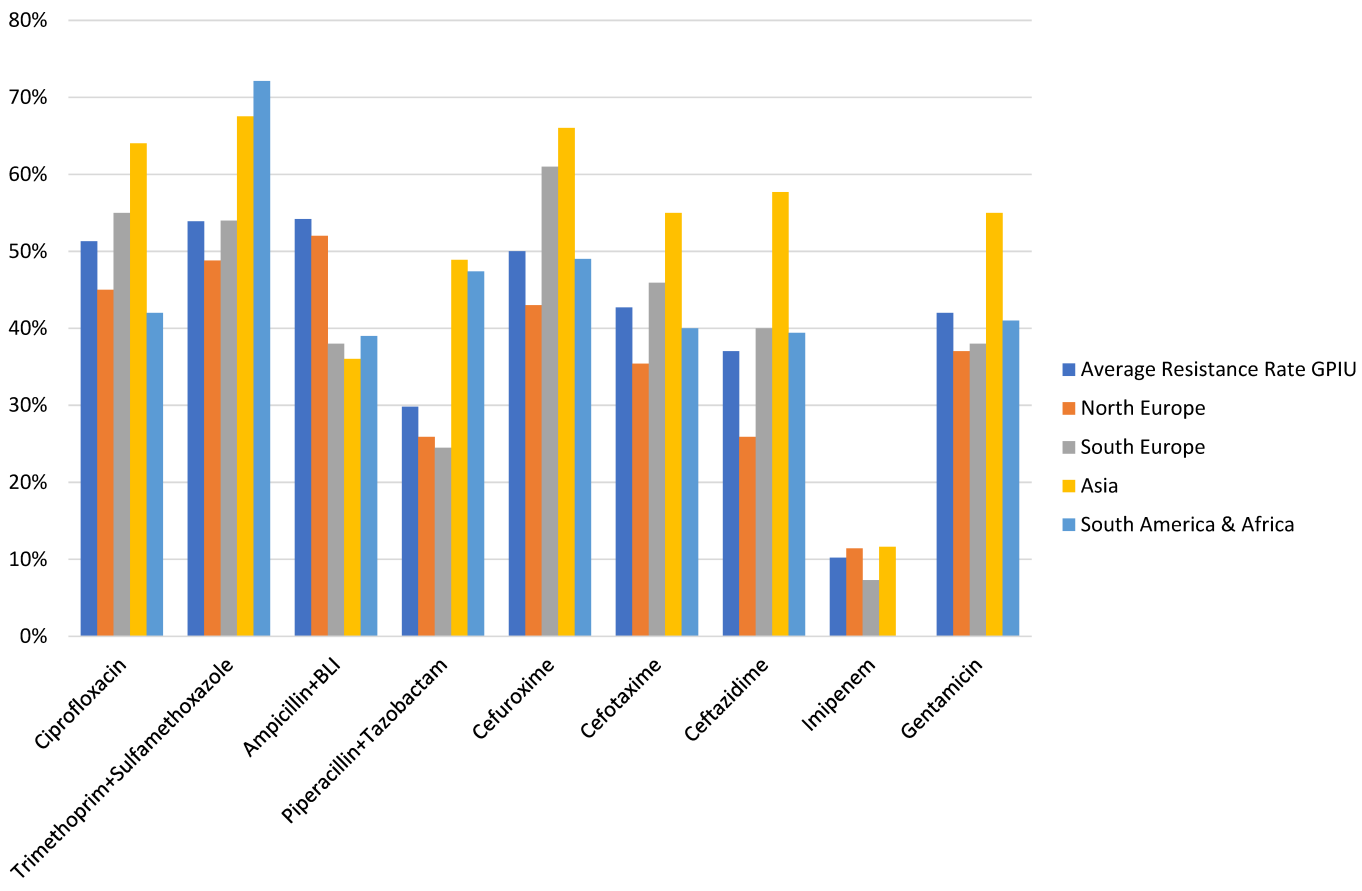
Resistance patterns to antibiotics in patients with HAUTIs from the GPIU study 2003–2013 [29]; BLI: betalactamase inhibitor

**Figure 5:**
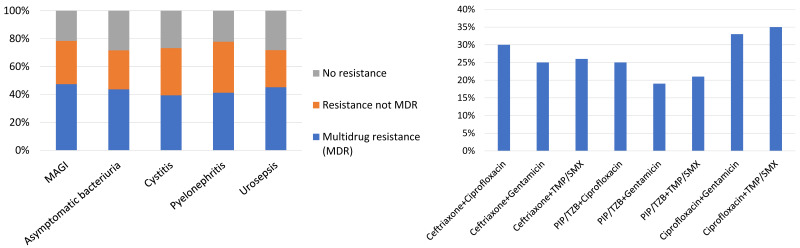
Resistance patterns to antibiotics in patients with HAUTIs from the GPIU study 2003–2013; left: prevalence of multidrug resistance (MDR) microorganisms; right: resistance rates for the main groups of antibiotics [29]; MDR: multidrug resistance, MAGI: male accessory gland infection, ASB: asymptomatic bacteriuria, Cefta: ceftriaxone, Cipro: ciprofloxacin, Genta: gentamicin, TMP/SMX: trimethoprim-sulfamethoxazole, Pip/Tzb: piperacillin/tazobactam

The isolation of extended-spectrum beta-lactamase (ESBL) producing bacteria is a significant concern, as the selection of adequate antibiotic treatment is a challenging task. In patients with HAUTIs hospitalized in a urology ward, a percentage of 27.8% ESBL-producing bacteria has been described [32], [45]. These data are in agreement with other general series, which report percentages between 15% and 44% in hospitalized patients [46]. The higher risk of isolation of multidrug-resistant pathogens in urological patients is related, among others, to a higher prevalence of urinary catheter carriers during hospitalization. Figure 4 (Fig. 4) and Figure 5 (Fig. 5) summarize the results regarding resistance in patients with HAUTIs from the GPIU study [20], [26], [28], [29].

The presence of different comorbidities is associated with a higher probability of isolation of ESBL-producing bacteria. Older age, male sex, diabetes mellitus, urinary or nasogastric catheter, previous admission or institutionalization in nursing homes, and previous urinary tract infections are risk factors for ESBL-producing bacteria isolation [10], [46], [47], [48]. A review carried out in Spain shows that age over 65 years, the presence of a urinary catheter, urological patients and previous treatment with quinolones are risk factors for the isolation of ESBL-producing microorganisms [49]. Interestingly, all these risk factors are included in the ORENUC classification as risk factors for a more severe outcome [21], [22]. Knowledge of epidemiological data about the resistance patterns of the area and the characteristics of the patient may minimize therapeutic failures and counteract the appearance of resistance [10], [31]. In case of suspicion of ESBL-producing bacteria, carbapenems are considered the treatment of choice due to their beta-lactam ring being more resistant to hydrolysis by ESBL enzymes [50], [51]. However, it should not be forgotten that mechanisms of resistance to carbapenemases are also emerging [52]. In case of UTIs without septicaemia due to ESBL-producing bacteria, it is appropriate to consider treatment with piperacillin/tazobactam, as cultures usually show susceptibility to this antibiotic [53], [54]. In recent years, due to their high sensitivities, the usefulness of fosfomycin or nitrofurantoin in order to avoid prescription of carbapenems [55], [56] in the first-line treatment of uncomplicated community-acquired urinary tract infections caused by ESBL has been proposed. Oral pivmecillinan (prodrug of mecillinan) may also be a suitable alternative in the management of uncomplicated UTIs due to ESBL-producing bacteria [57]. Therefore, it must be kept in mind that it is not considered an adequate antibiotic in the case of complicated urinary tract infections or hospitalized patients [50], [58]. The development of infections by ESBL-producing bacteria not only has consequences on morbidity and mortality; it also implies an increase in healthcare costs. It is estimated that infection by ESBL-producing bacteria carries an extra cost of $16,450 due to a longer stay [51], [59].

### Patient profiles in urology

Performing a surgical procedure is a risk factor for the subsequent development of an infection. Besides, a high percentage of the patients admitted to a urology ward will undergo some type of surgery during admission. Therefore, among measures to reduce the development of UTIs are adequate antibiotic prophylaxis, maintaining sterility in the surgical field and in the hospitalization unit, and removal of urinary catheters as early as possible [27], [60]. When selecting prophylaxis, factors such as the type of surgery, but also the duration of surgery, the degree of invasiveness of the technique, the general condition of the patient (presence of comorbidities, nutritional status, and age) must be taken into account [61]. The review by Cai et al., carried out in a urology department in an Italian tertiary hospital, has shown that correct use of prophylaxis allowed a reduction in drug costs, from €76,980 in the period from 2008 to 2010, to €36,700 in the period from 2011 to 2013. *E. coli* resistance to piperacillin-tazobactam, gentamicin and ciprofloxacin also decreased [62]. Endourological surgery is the type of urological surgical procedure most frequently performed, accounting for up to 54.5% of the surgeries carried out in urology [32]. It is a group of patients in whom there are usually two risk factors for HAUTIs: on the one hand, the surgical procedure itself with access to the urinary tract and, on the other hand, the need in most cases for catheterization of the urinary tract in the postoperative period [18]. Most of the series have been conducted evaluating patients undergoing transurethral resection of the prostate (TURP) with UTI incidences after surgery between 2% and 6% [63], [64]. Another study published by Pestalozzi et al., which shows similar figures for UTIs after TURP, also describes a rate of 3% after transurethral resection of the bladder [65]. Regarding other types of endoscopic surgery, Sohn et al. describe 3.8% infectious complications after instrumentation of the upper urinary tract and the risk factors for the development of HAUTIs were the existence of pre-surgical bacteriuria, hydronephrosis or presence of a urinary diversion catheter (urinary catheter, double J catheter, or nephrostomy) [66]. Factors associated with a higher prevalence of HAIs after endoscopic surgery are a longer surgical time and bleeding [67]. The first point to note regarding the microbiological data after endoscopic surgery is the high percentage of asymptomatic bacteriuria, which reaches figures of up to 60% [63], [67]. Regarding the resistance rates, it is worth highlighting the high resistance to quinolones, which is above 50% for *E. coli*, *Pseudomonas aeruginosa* and *Enterococcus* spp.

Urinary tract infections in patients with a urinary catheter is of particular relevance in urology due to the high percentage of patients who require a urinary catheter. It is estimated that 12–16% of hospitalized patients carry a urinary catheter, regardless of the unit. This percentage rises to 67–70% in Urology Services. 70–80% of UTIs related to health care are associated with a urinary catheter [12]. The Centers for Disease Control and Prevention (CDC) estimates that up to 139,000 urinary catheter-associated UTIs occurred in the United States in 2007 [18]. Furthermore, UTIs related to carrying a urinary catheter is associated with higher morbidity, mortality, and costs. Also, each episode of UTI associated with a urinary catheter has a cost of $600, which amounts to $2,800 if there is sepsis and the infection spreads to the bloodstream. Risk factors described for UTIs in bladder catheter holders include a longer catheter time, female sex, older age, and not using a closed drainage system [12]. The urinary catheter is the most frequently used type of catheter, 66% in urology departments. Sometimes it is associated with carrying other types of urinary diversion, preferably double J stents. When analyzed in isolation and not associated with other types of catheters, it is carried by 57% of admitted patients in a urology department [68].

The presence of a urinary catheter before admission is related to an increased risk of HAUTIs [69]. It is well established that longer catheterization time is related to a higher incidence of infection [2]. Other factors associated with an increased risk of HAUTIs described in the literature are debilitating chronic diseases and comorbidities, immunosuppression and UTIs in the previous months as described in the ORENUC system [22], [70], [71]. The most commonly isolated pathogen is *E. coli*, which represents 26–65% of positive cultures [14], [72]. In addition to *E. coli*, there is a high prevalence of infections caused by other *Enterobacterales*, especially in patients with chronic catheterization [73]. However, infections related to *Enterococcus faecalis* and *Pseudomonas aeruginosa* are also frequent, and represent 15.5% and 14.1% of the isolated germs, respectively [74]. These results are similar to those published by Wazait et al. in a study carried out in the United Kingdom; *Enterococcus* represented up to 22% of positive cultures and *Pseudomonas aeruginosa* around 11% [72]. The main concern in the management and prevention of HAUTIs is the high rates of *E. coli* resistance to commonly used antibiotics such as fluoroquinolones (57.9%). Other pathogens such as *Klebsiella* spp., *Enterococcus* spp., and *Pseudomonas aeruginosa* have quinolone resistance rates around 60% [45], [74]. Another point worthy of attention is the percentage of ESBL-producing bacteria. Results from southern European countries show ESBL-producing bacteria rates of up to 59% [25], [75], [76]. A key point in the management of patients with urinary catheters is to prevent the development of UTIs. Therefore, urinary catheters should be removed as early as possible, and their management must be optimized. Among preventive measures, the use of closed drainage systems stands out [77]. Proper care of urinary catheters is essential since it has been observed that UTIs associated with carrying a urinary catheter can be reduced by 53% [12].

Catheters used for diversion of the upper urinary tract, such as double J stent and nephrostomy tubes are also associated with a high incidence of HAUTIs and isolation of ESBL and multidrug-resistant microorganisms [32], [68]. Double J stents are used in case of hydronephrosis, after surgical procedures for the treatment of renal or ureteral stones, after pyeloplasty and ureteral surgery [78]. These types of catheters are not exempt from morbidities, such as lower abdominal pain, dysuria, hematuria, migration, and development of urinary tract infections. The pathogenesis of the development of UTIs associated with a double J catheter is related to the fact that any type of catheter is colonized by bacteria that can cause an infectious process [79]. The use of double J catheters is frequent in urological patients. Akay et al. have reported that 44–68% of double J stents are colonized by the time the ureteral stents are removed [80]. Furthermore, urinary tract infection is the most frequent complication associated with carrying this type of catheter, with a published incidence of 5.4% [80]. Among the risk factors that have been described for urinary tract infection in double J carriers are female sex, pregnancy, presence of comorbidities such as diabetes mellitus, kidney failure and long-term double J catheter use [80], [81]. The most frequently isolated pathogens in this type of patients are *Enterococcus*, *E. coli*, *Pseudomonas* and *Candida albicans* [81], [82].

A percutaneous nephrostomy tube is a diversion catheter of the upper urinary tract used for the first time in 1954 [83]. The indications for its placement are urinary tract obstruction caused by stones, clots, malignant pathologies or ureteral strictures. They are also used in association with some therapeutic procedures, such as percutaneous nephrolithotomy [84]. The incidence of infectious complications in patients with nephrostomy is estimated to be around 3.5%, with a probability of sepsis of 1% [83]. The risk factors described for the development of infections in patients with nephrostomy are advanced age, diabetes mellitus, bladder dysfunction, presence of a previous urinary catheter, uretero-intestinal anastomosis, manipulation of the catheter, bacteriuria, and presence of stones [85]. Nephrostomy tubes are placed through a percutaneous access. For this reason, it has been suggested that the pathogenic mechanism for the development of infections is often related to microorganisms from the skin that colonize the catheter during insertion or manipulation [83]. It is worrying that among patients with nephrostomy who seek medical care at the emergency room with a urinary tract infection, 42.9% of those with positive cultures for *E. coli* show ESBL-producing germs. In the case of *Klebsiella*-positive cultures, multidrug-resistant microorganisms may reach up to 85% [45], [68].

## Conclusions

HAUTIs are the main types of HAIs in patients admitted in a urology ward. Risk factors related to HAUTIs are prior urinary tract infection, an indwelling urinary catheter and comorbidities such as immunosuppression, all of which are included in the ORENUC system. Although *E. coli* is the most frequently isolated pathogen, other microorganisms such as *Klebsiella*, *Enterococcus* and *Pseudomonas aeruginosa* are commonly found. High resistance rates are reported, such as ESBL-producing bacteria. Therefore, antibiotic stewardship plays a crucial role in the control of infections. Treatment must be tailored, considering individual risk factors and presumed etiology.

Observational studies and continuous monitoring of HAUTIs are recommended measures to reduce the incidence of infections and optimize their management.

## Note

This article will also be published as a chapter of the Living Handbook “Urogenital Infections and Inflammations” [86].

## Acknowledgments

We acknowledge the effort and collaboration of all members of the ESIU (European Association of Urology Section of Infections in Urology) in the prevention of uri-nary tract infections and the optimization of their management.

## Authors’ ORCIDs

- José Medina-Polo: 0000-0003-3626-8669
- Kurt G. Naber: 0000-0003-1304-5403
- Truls E. Bjerklund Johansen: 0000-0003-3490-6460

## Competing interests

The authors declare that they have no competing interests.

## Figures and Tables

**Table 1:**
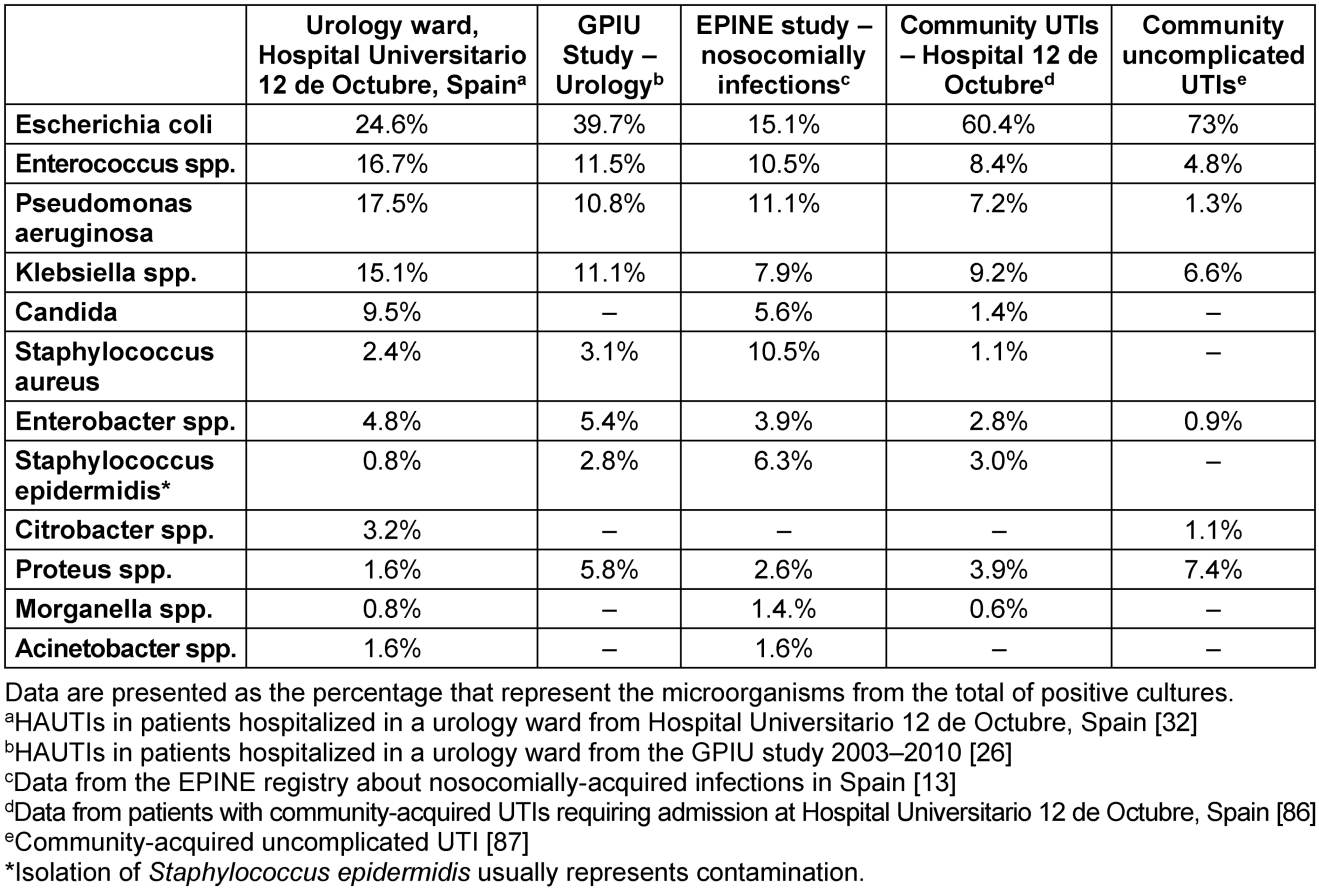
Microorganisms isolated in patients with UTIs (HAUTIs, community-acquired and uncomplicated)

**Table 2:**
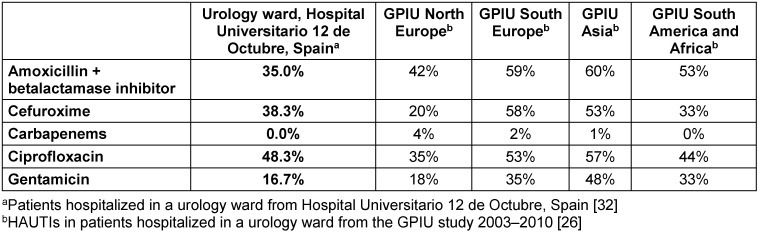
E. coli resistance rate in patients with HAUTIs hospitalized in urology departments

**Table 3:**
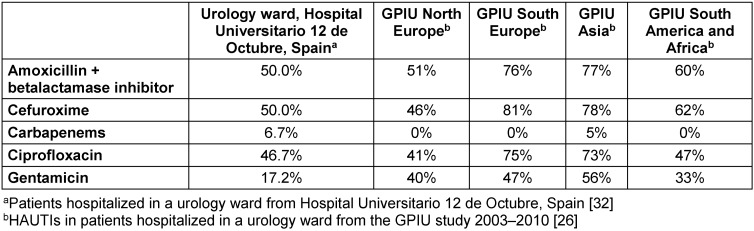
Klebsiella spp. resistance rate in patients with HAUTIs hospitalized in urology departments

**Table 4:**
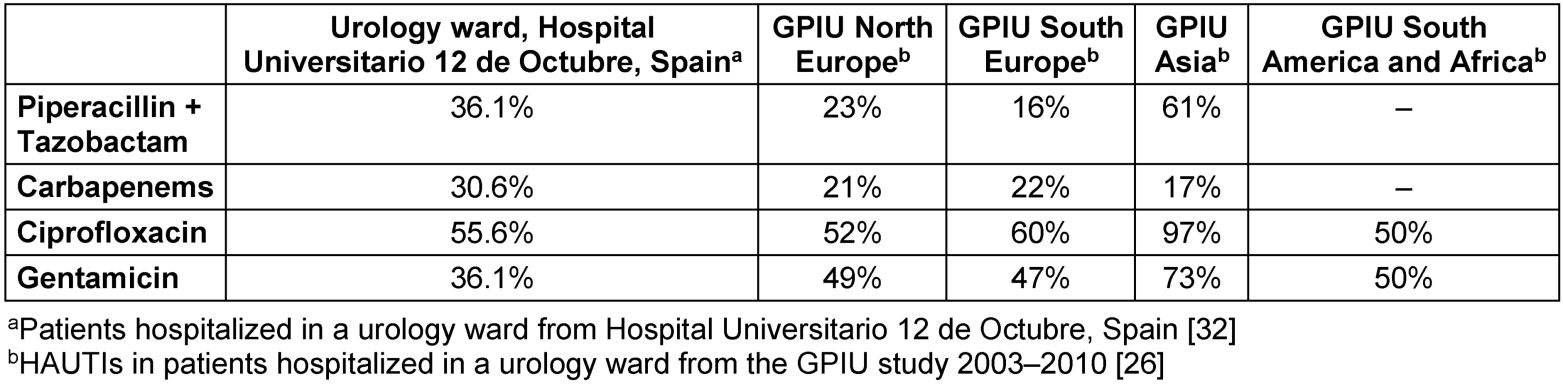
Pseudomonas aeruginosa resistance rate in patients with HAUTIs hospitalized in urology departments

**Table 5:**
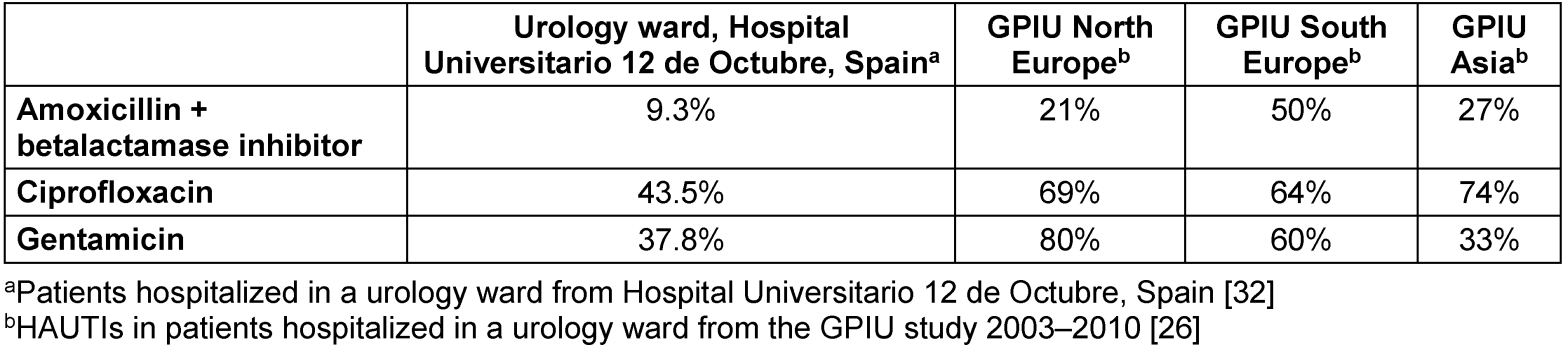
Enterococcus spp. resistance rate in patients with HAUTIs hospitalized in urology departments
